# BACH1 regulates the proliferation and odontoblastic differentiation of human dental pulp stem cells

**DOI:** 10.1186/s12903-022-02588-2

**Published:** 2022-11-24

**Authors:** C. Liu, J. Yu, B. Liu, M. Liu, G. Song, L. Zhu, B. Peng

**Affiliations:** The State Key Laboratory Breeding Base of Basic Science of Stomatology (Hubei-MOST) and Key Laboratory of Oral Biomedicine Ministry of Education, School and Hospital of Stomatology, Wuhan University, Wuhan, People’s Republic of China

**Keywords:** BTB and CNC homology 1, BACH1, Human dental pulp stem cell, Odontoblastic differentiation, Odontogenesis

## Abstract

**Background:**

The preservation of biological and physiological vitality as well as the formation of dentin are among the main tasks of human dental pulp for a life time. Odontoblastic differentiation of human dental pulp stem cells (hDPSCs) exhibits the capacity of dental pulp regeneration and dentin complex rebuilding. Exploration of the mechanisms regulating differentiation and proliferation of hDPSCs may help to investigate potential clinical applications. BTB and CNC homology 1 (BACH1) is a transcription repressor engaged in the regulation of multiple cellular functions. This study aimed to investigate the effects of BACH1 on the proliferation and odontoblastic differentiation of hDPSCs in vitro.

**Methods:**

hDPSCs and pulpal tissues were obtained from extracted human premolars or third molars. The distribution of BACH1 was detected by immunohistochemistry. The mRNA and protein expression of BACH1 were examined by qRT-PCR and Western blot analysis. *BACH1* expression was regulated by stable lentivirus-mediated transfection. Cell proliferation and cell cycle were assessed by cell counting kit-8 assay, 5-Ethynyl-2'-deoxyuridine assay and flow cytometry. The expression of mineralization markers, alkaline phosphatase (ALP) activity and alizarin red S staining were conducted to assess the odontoblastic differentiation ability.

**Results:**

BACH1 expression was stronger in the odontoblast layer than in the cell rich zone. The total and nuclear protein level of BACH1 during odontoblastic differentiation was downregulated initially and then upregulated gradually. Knockdown of *BACH1* greatly inhibited cell proliferation, arrested cell cycle, upregulated the heme oxygenase-1 (HO-1) expression and attenuated ALP activity, decreased calcium deposits and downregulated the expression of mineralization markers. Treatment of Tin-protoporphyrin IX, an HO-1 inhibitor, failed to rescue the impaired odonto/osteogenic differentiation capacity. Overexpression of *BACH1* increased cell proliferation, ALP activity and the expression of mineralization markers.

**Conclusions:**

Our findings suggest that BACH1 is an important regulator of the proliferation and odontoblastic differentiation of hDPSCs in vitro. Manipulation of BACH1 expression may provide an opportunity to promote the regenerative capacity of hDPSCs.

**Supplementary Information:**

The online version contains supplementary material available at 10.1186/s12903-022-02588-2.

## Background

The main functions of human dental pulp include the maintenance of biological and physiological vitality and the production of dentin throughout the life [1]. Although dentin can be synthesized by odontoblasts throughout life as long as the tooth remains alive, human dental pulp is vulnerable to injury, inflammation, microbial pathogens and other stimuli that may cause pulp necrosis and degeneration. When met with an appropriate stimulus, such as dental decay, inflammation and trauma, odontoblasts and odontoblast-like cells from dental pulp might form reparative dentin [2–4].

Human dental pulp stem cells (hDPSCs) can repair pulp tissue injury partially or completely, which are isolated and derived from normal pulp tissue and have the ability to differentiate into odontoblasts, osteoblasts, adipocytes, chondrocytes and neural cells under proper environmental conditions or certain induction [1, 5, 6]. Through teeth extraction, hDPSCs are readily available and possess strong abilities of self-renewal, proliferation and multilineage differentiation. Due to these advantages, tissue engineering and regenerative medicine have placed a significant amount of attention on hDPSCs [7–9]. In these fields, odontoblastic differentiation of hDPSCs exhibits the capacity of dental pulp regeneration and dentin complex rebuilding [10–12]. Nevertheless, the mechanisms under oriented differentiation of hDPSCs are not yet fully understood.

BTB and CNC homology 1 (BACH1), a transcription repressor of the Cap ‘n’ Collar and basic region leucine zipper family (CNC-bZip), forms heterodimers with the small Maf proteins such as MafK. Then the BACH1-Maf heterodimers bind to Maf recognition elements (MAREs) in the gene promoters [13]. BACH1 is widely expressed in mammalian tissues and engaged in the regulation of cell proliferation and differentiation, cell cycle, redox balance and other cellular functions [14–16]. Muscle regeneration was impaired in *BACH1*-deficient mice, and the proliferation and differentiation markers expression of myoblasts were inhibited due to the *BACH1* silencing [17]. Similarly, RANKL-mediated osteoclastogenesis was also attenuated [18]. The down regulation of BACH1 promoted the angiogenic activity of human microvascular endothelial cells [19]. Hence, the role of BACH1 in differentiation varies across different cells and species.

Although multiple impacts of BACH1 on molecule mechanisms have been investigated, the role of BACH1 in the proliferation ability and odonto/osteogenic potential of hDPSCs remains unclear. In the present study, we aimed to determine the distribution of BACH1 in healthy human dental pulp tissues, investigate the expression patterns of BACH1 during odontoblastic differentiation, and explore the role of BACH1 in the proliferation and odontoblastic differentiation of hDPSCs in vitro.

## Methods

### Materials

OriCell Supplement for Human Related Stem Cells Adipogenic Differentiation kit and OriCell Supplement for Human Related Stem Cells Chondrogenic Differentiation kit were purchased from Cyagen (Guangzhou, China). PE-CD73 (344003), PE-CD90 (328109) and PE-CD146 (361005) antibodies were form BioLegend (San Jose, CA, USA). PE-CD45 (200349) and APC-CD34 (509459) were obtained from Tonbo (San Diego, CA, USA). Ultra Sensitive™ SP (Mouse/Rabbit) IHC Kit and DAB Detection Kit were obtained from Maixim (Fuzhou, China). Triton X-100, 4,6-diamidino-2-phenylindole (DAPI), radio-immunoprecipitation assay (RIPA) buffer, Nuclear and Cytoplasmic Protein Extraction Kit, BCIP/NBT Alkaline Phosphatase Color Development Kit, Alkaline Phosphatase Assay Kit, BCA Protein Assay Kit and Proliferation Kit with Alexa Fluor 594 were purchased from Beyotime Biotechnology (Beyotime, Shanghai, China). Phosphate-buffered saline (PBS), minimum essential medium alpha (α-MEM), foetal bovine serum (FBS); penicillin–streptomycin were obtained from HyClone Laboratories (HyClone, Logan, UT, USA). Beta-glycerophosphate, ascorbic acid, dexamethasone, methylpyridinium and chloride FITC conjugated phalloidin are all from Sigma-Aldrich (Sigma, Basel, Switzerland). Protease inhibitor cocktails and phosphatase inhibitor cocktails, polyvinylidene fluoride membranes and the Transcriptor High Fidelity cDNA Synthesis Kit were obtained from Roche (Indianapolis, IN, USA). BIO-RAD Real-time PCR System and Trans-Blot Turbo Transfer System were obtained from Bio-Rad (Hercules, CA, USA). BACH1 (14018-1-AP), HO-1 (66743-1-Ig), Lamin B1 (66095-1-Ig), α-Tubulin (66031-1-Ig), CoraLite488-Goat-anti-Rabbit IgG (H + L) and CoraLite594-goat-anti-Mouse IgG (H + L) were purchase from Proteintech (Chicago, IL, USA). BACH1 (SC-271211) were from Santa Cruz Biotechnology (Santa Cruz, CA, USA). Dentin sialophosphoprotein (DSPP; A8413) and dentin matrix protein 1 (DMP1; A16832) were from Abclonal Technology (Abclonal, Wuhan, China). Runt-related transcription factor 2 (RUNX2; 12556), was purchased from Cell Signaling Technology (CST, Beverly, USA). GAPDH (PMK053S) was from Bioprimacy (Wuhan, China). Cy3-conjugated-goat-anti-mouse IgG (AS1111) was obtained from Aspen Biotechnology (Wuhan, China). Tin-protoporphyrin IX (SnPP, 10 µM; HY-101194) was obtained from Med Chem Express (MCE, Monmouth Junction, NJ, USA). Cell Counting Kit-8 (CCK-8) was obtained from Dojindo (Kumamoto, Japan). Collagenase type I was from Invitrogen (Carlsbad, CA, USA). MAG Viral Nucleic Acid Purification Kit was purchased from Axygen Scientific (Axygen, Union City, California, USA). Enhanced Chemiluminescence Western Blotting Substrate was from Thermo Scientific (Thermo, Waltham, MA, USA). ChamQ SYBR Color qPCR Master Mix kit was from Vazyme (Nanjing, China). Alizarin Red S solution (pH = 4.2) was from Solarbio (Beijing, China).

### Primary cell culture and treatments

Extracted healthy third molars (n = 12) were obtained from donors (18–22 years of age). Pulp tissues from freshly extracted healthy teeth were gently separated from the teeth and cut into tiny pieces (0.5–1.0 mm^3^), digested in a solution of 6 mg/mL collagenase type I for 1 h at 37 ℃, and then cultured in α-MEM with 100 U/mL penicillin–streptomycin plus 10% FBS in a humidified atmosphere of 5% CO_2_ at 37 ℃. The cells between the third and sixth passages were used.

### Multi‑lineage differentiation in vitro

For odontoblastic differentiation, 2 × 10^5^ per well hDPSCs were cultured in 6-well plates. At 70–80% confluence, cells were cultured with odonto/osteogenic induction medium (OM) containing 10% FBS, 100 U/mL penicillin–streptomycin, 10 mmol/L beta-glycerophosphate, 50 µg/mL ascorbic acid and 10 nmol/L dexamethasone. The culture medium was changed every 3 days, and the samples were obtained after 18 days. The cells and extracellular calcium deposits were then stained with alizarin red S staining solution. The adipogenic differentiation medium and the chondrogenic differentiation medium were purchased from Cyagen and the components of these induction medium were shown in Additional file 2: Tables S1–S3. For adipogenic induction, the cells were cultured in 12-well plates at the density of 5 × 10^4^ per well and the medium was changed regularly according to the manufacturer's recommendations. After 1 day of incubation with adipogenic differentiation maintenance medium, the medium was changed to adipogenic differentiation induction medium and incubated for 3 days. The medium was changed at the above frequency for 14 days. Cells were fixed and stained with oil red O staining solution on day 14. For chondrogenic differentiation, cells were resuspended in chondrogenic differentiation medium and the cell density was adjusted to 1.0–2.0 × 10^7^ cells/mL. 20 µL cell suspension was pipetted into the center of each well of a 24-well plate. The hDPSCs were incubated for 2.5 h at 37 °C to adhere and 1 mL of chondrogenic differentiation medium was added to each well after adherence, and the medium was changed every 3 days. After 21 days, pellets were fixed, paraffin-embedded and sectioned. The sections were stained with alcian blue staining solution.

### Flow cytometry analysis of stem cell surface markers

hDPSCs were digested with trypsin and resuspended in PBS containing 2% FBS to the cell density of 1 × 10^7^ cells/mL. Added 5 µL of coupled antibody to 95 µL cell suspension and incubated for 1 h at 4 °C, protected from light. One sample without the addition of antibody was used as a negative control. After cells were washed twice and re-suspended with PBS, the samples were analyzed by flow cytometry (FACS Calibur, Becton Dickinson, Franklin Lakes) and CytExpert Software (Beckman Coulter, Brea, CA, USA).

### Reverse transcription and quantitative real-time polymerase chain reaction (qRT-PCR)

After seeding cells into 6-well plates at 2 × 10^5^ per well in control and experimental group, cellular RNA was extracted from cultured hDPSCs by using an RNA isolation kit and cDNA was synthesized from 1 µg of total RNA by using the Transcriptor High Fidelity cDNA Synthesis Kit. qRT-PCR was performed in triplicates for each sample by using ChamQ SYBR Color qPCR Master Mix kit with a BIO-RAD Real-time PCR System. All primers were synthesized by Sangon (Shanghai, China). *Glyceraldehyde 3-phosphate dehydrogenase* (*GAPDH*) was used as a housekeeping gene to normalize mRNA expression. The gene-specific primer sequences used in this study are shown in Additional file 2: Table S4. The relative expression of genes of interest was estimated using the Δδthreshold cycle (Ct) method.

### Western blot analysis

Total lysates were obtained from cultured hDPSCs by using RIPA buffer containing the protease inhibitor cocktails and phosphatase inhibitor cocktails. The cytosolic and nuclear fractions were prepared using Nuclear and Cytoplasmic Protein Extraction Kit in line with the manufacturer’s instructions at the appropriate point of time. The purity of nuclear fractions was checked by anti-Lamin B1 (1:1000) and anti-α-Tubulin antibodies (1:1000). Protein concentrations were quantified using the BCA Protein Assay Kit. For protein separation, equal amounts of protein were loaded onto a 10% sodium dodecyl sulfate–polyacrylamide gel and then transferred on polyvinylidene fluoride membranes using the Trans-Blot Turbo Transfer System. After being blocked with 5% skim milk in Tris-buffered saline with 0.05% Tween-20 (TBST) for 1 h at room temperature, the membrane was incubated with primary antibodies overnight at 4 °C. The membrane was washed thrice with TBST and then cultured with the corresponding secondary antibodies (1:3000; CST) for 1 h at room temperature. The protein was visualized using Enhanced Chemiluminescence Western Blotting Substrate after three washes for 10 min in TBST. Specific primary antibodies including BACH1 (1:500; Santa Cruz), GAPDH (1:8000), DSPP, DMP1, RUNX2, (all diluted at 1:1000). The protein expression level was quantified using Image Pro Plus 6.0 (Media Cybernetics).

### Alkaline phosphatase (ALP) activity assay

Samples were collected at 3, 5 or 7 days. ALP activity was analyzed using BCIP/NBT Alkaline Phosphatase Color Development Kit and Alkaline Phosphatase Assay Kit following the manufacturer’s instructions. Protein concentrations were also tested using the BCA method as the standard. Absorbance was measured at 405 and 562 nm for ALP activity assay and protein quantification, respectively.

### Alizarin red S (ARS) staining assay

After 14 or 21 days of differentiation induction, cells in six-well plates were rinsed with PBS and fixed in 4% Paraformaldehyde for 15 min at room temperature. Subsequently, the matrix and cells were stained with 1% alizarin red S solution for 30 min at 37 ℃, washed thrice per 5 min with deionized water remove unbound stains, cells were air-dried before being photographed. For quantitative evaluation, stain was dissolved in 10% methylpyridinium chloride for 5 min at room temperature. 1 mL supernatant from each well was obtained and the absorbance of supernatant at 562 nm was measured.

### Cellular immunofluorescence

The cells were seeded on sterile glass cover slips at a density of 1 × 10^4^ cells per cover. After cultured according to the study design, the cells were fixed with 4% paraformaldehyde for 15 min, washed thrice with PBS, treated with 0.1% Triton X-100 for 5 min, blocked with 10% non-immune goat serum for 1 h at room temperature, and then incubated with primary antibody BACH1 (1:100; Santa Cruz) in 4 ℃ overnight. After being washed twice, the cells were incubated with Cy3-conjugated-goat-anti-mouse IgG (1:200) for 1 h at 37 ℃. The cytoskeleton and nuclei were stained with 5 µg/mL FITC conjugated phalloidin for 40 min, and DAPI for 5 min at 37 ℃. The coverslips were then overlaid on a microscope slide with the embedding medium. Afterward, cells were observed and photographed using a confocal microscope (Olympus Corporation, Japan).

### Lentivirus transduction

Four types of lentivirus plasmids LV-shRNA-BACH1 (LV-shBACH1), LV-Negative Control (LV-NC, blank vector), Vector and pCMV-BACH1, which were obtained from Genechem (Shanghai, China) were transfected into hDPSCs to establish the knockdown and overexpression groups according to manufacturer’s guidelines. The culture medium was changed upon transfection for 48 h with 2 µg/mL puromycin to select cells, and cells were collected to carry out subsequent analyses. The cell transfection efficiency and knockdown or overexpression efficiency were evaluated by microscopic fluorescence, qRT-PCR and Western blot analysis.

### Cell proliferation assay

Cell proliferation was evaluated using the CCK-8 based on the manufacturer’s instructions. Briefly, cells (8 × 10^3^) were seeded in each well of a 96‐well plate, 10 µL CCK-8 solution was added into each well with serum-free α-MEM, and the samples were incubated at 37 °C, and protected from light for 1 h. Optical density was measured at 450 nm. The assay was conducted at 0, 24, 48 and 72 h to generate a growth curve.

### 5-Ethynyl-2'-deoxyuridine (EdU) assay

According to the manufacturer’s instructions, the Proliferation Kit with Alexa Fluor 594 (Beyotime) was used to evaluate the effect of *BACH1* downregulation on hDPSCs proliferation capacity. After being transfected into 12-well plate for 48 h, hDPSCs reached 50–70% confluence and were EdU labelled for 2 h. Then, click reaction buffer and Hoechst 33342 staining was carried out. Fluorescence microscopy (Olympus) was used to photograph and count the cells.

### Cell cycle assay

The cells were cultured in a 6-well plate at a density of 5 × 10^4^ cells/well, and then transfected for 48 h. A cell cycle staining Kit were used for the different phases of cell-cycle analysis. According to the directions, the cells were digested, fixed and stained with the PI staining solution with RNase A. After incubated for 30 min in the dark at room temperature, stained samples were tested by flow cytometry (FACS Calibur) and analyzed by ModFit LT software 4.

### Immunohistochemistry

Freshly extracted healthy adults’ teeth (third molar or pre molar, *n* = 7) were obtained and fixed with 4% paraformaldehyde immediately at 4 ℃ for 48 h, and then rinsed, decalcified with 10% EDTA, dehydrated, and embedded in paraffin. Serial sections of 5 µm thickness were cut in the horizontal direction and subjected to hematoxylin–eosin (H&E) staining. Immunohistochemistry was conducted using the Ultra SensitiveTM SP (Mouse/Rabbit) IHC Kit and DAB Detection Kit according to the manufacturer’s instructions. Rabbit polyclonal antibodies against BACH1 (1:150; Proteintech) were used as the primary antibodies for 24 h at 4 °C. PBS, a primary antibody diluting liquid, was used as a negative control. Counterstaining was performed with haematoxylin for light microscopy (Leica, Wentzler, Germany).

### Double immunofluorescence labelling

The tooth sections were double-labeled with BACH1 and HO-1 as the previous approach [20]. Briefly, after deparaffinization and rehydration, the sections were treated with EDTA antigen retrieval solution was used at 95 ℃ for 30 min. Rehydrated sections were incubated with 2.5% bovine serum albumin for 1 h to eliminate nonspecific staining and then incubated overnight with BACH1 rabbit polyclonal antibody (1:150; Proteintech) and HO-1 mouse monoclonal antibody (1:50; Proteintech) at 4 ℃. The sections were washed and incubated with the secondary fluorescein CoraLite488-Goat-anti-Rabbit IgG (H + L) (1:100; Proteintech) and CoraLite594-goat-anti-Mouse IgG (H + L) antibodies (1:250; Proteintech) at 37 ℃ for 1 h, and the nuclei were stained with DAPI for 5 min. Finally, the slides were analysed by fluorescent microscopy (Leica). Three different regions were selected randomly in odontoblast layer and cell-rich zone to count the number of DAPI, BACH1-positive or HO-1-positive cells by Image Pro Plus 6.0 (Media Cybernetics, Silver Spring, USA). Positive cell rate = the number of positive cells/the number of total cells (DAPI) × 100%. Each field was photographed at 400 × magnification under different wave length [21].

### Statistical analysis

Results are presented as mean ± the standard error of the mean (SEM) from at least three replicates. Statistical analysis was performed using Graphpad Prism software (GraphPad Software, San Diego, California, USA), by Student’s *t*-test or one-way analysis of variance (ANOVA) followed by Dunnett’s post hoc test or Tukey's multiple comparisons test after homogeneity of variance test. *P* < 0.05 was considered statistically significant.

## Results

### BACH1 was mainly located in the odontoblast layer of human dental pulp tissues

**Fig. 1:**
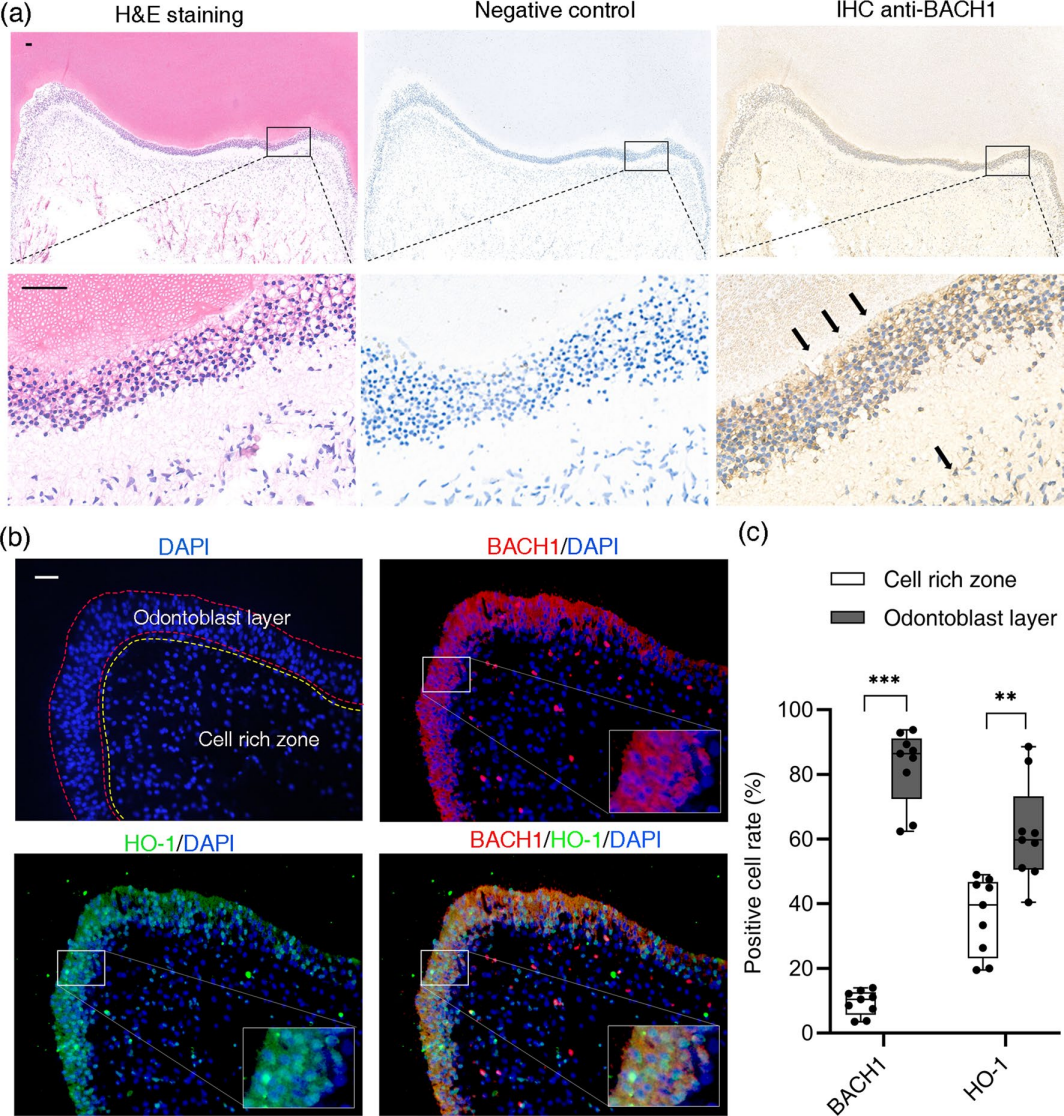
BACH1 was mainly located in the odontoblast layer of human dental pulp tissues. **a** Immunohistochemical staining was performed to show the BACH1 expression in healthy human dental pulp tissues; *arrowheads* indicate BACH1 expression. Scale bars = 50 µm. **b** BACH1 (red) and HO-1 (green) expression was detected by double immunofluorescence staining. Blue, DAPI-stained cells. **c** Positive cell rate analysis indicated that the expression of BACH1 and HO-1 increased in the odontoblast layer (red) compared with the cell rich zone (yellow). Scale bar = 20 µm. Data were presented as the mean ± standard error of the mean (n = 7). **P < 0.01, ***P < 0.001

The expression of BACH1 was shown throughout the dental pulp tissues, mainly in the odontoblast layer adjacent to the dentin and in fibroblast-like cells in the cell rich zone (Fig. 1a). No staining was observed with control IgG (Fig. 1a). The BACH1-positive cell rate (82.44 ± 3.86%) in the odontoblast layer was higher than that (9.345 ± 1.279%) of the cell rich zone (*P* < 0.001). HO-1 is also intensively expressed in the odontoblast layer (*P* < 0.05) (Fig. 1b, c).

### Characterization of hDPSCs

**Fig. 2:**
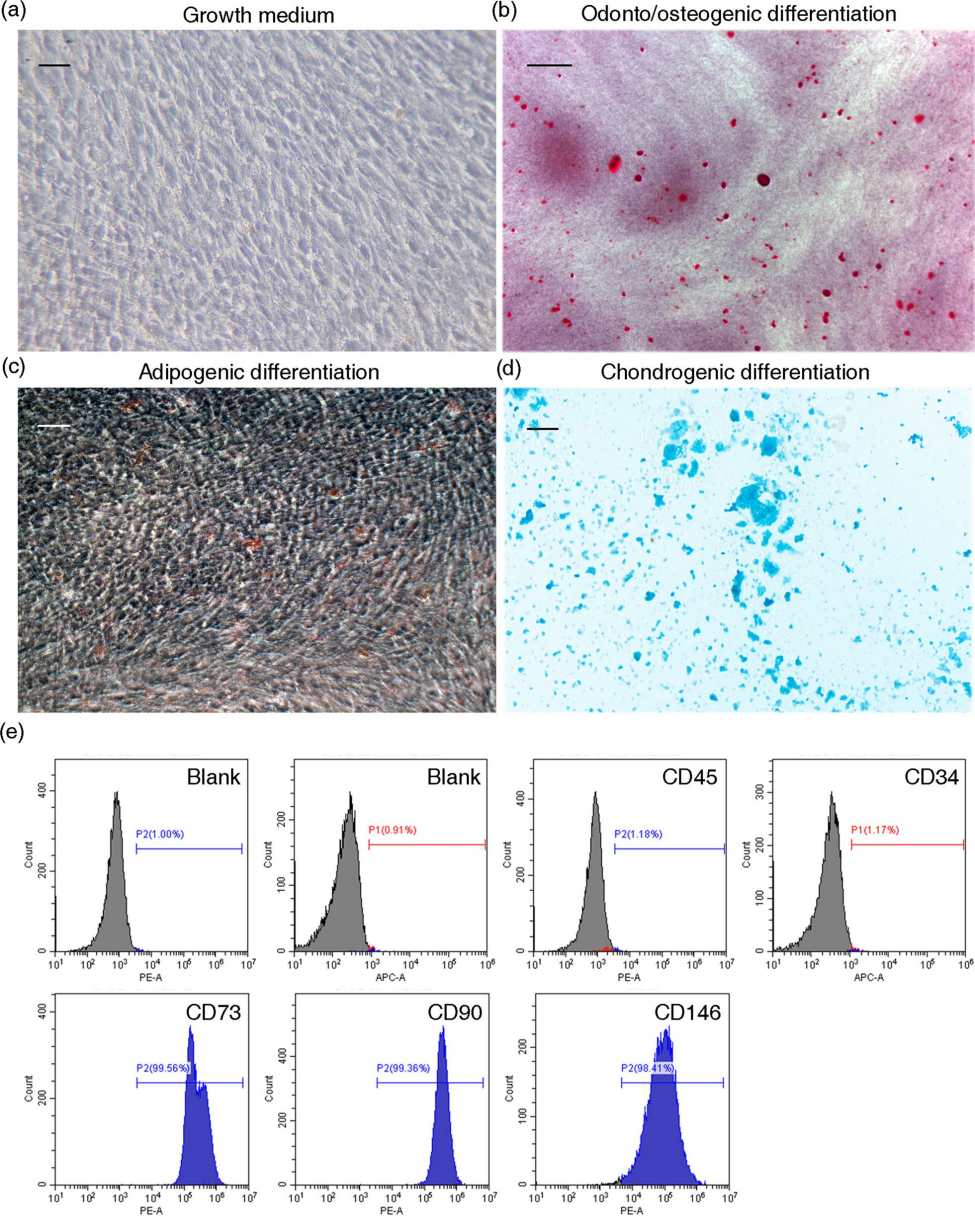
Characterization of hDPSCs. **a** hDPSCs cultured in growth medium for 21 days, scale bar = 20 µm. **b** Alizarin red S staining of hDPSCs cultured in odonto/osteogenic medium for 18 days, scale bar = 500 µm. **c** Oil red O staining of hDPSCs cultured in adipogenic medium for 14 days, scale bar = 20 µm. **d** Alcian blue staining of hDPSCs cultured in chondrogenic medium for 21 days, scale bar = 20 µm. **e** Flow cytometry exhibited that the expression of CD73, CD90, CD146 were positive in hDPSCs, which were negative against CD34 and CD45

Cells were characterized by multiple lineage differentiation tests and flow cytometry of cell surface markers. hDPSCs were cultured in growth medium (Fig. 2a) or under inductive conditions in which positively stained with alizarin red S (odontoblastic differentiation, Fig. 2b), oil red O (adipogenic differentiation, Fig. 2c), and alcian blue (chondrogenic differentiation, Fig. 2d), exhibiting heterogeneous differentiation ability. Flow cytometry analysis indicated that hDPSCs expressed CD73, CD90, CD146 highly (> 90%). In contrast, the hematopoietic cell markers CD34 and CD45 had a low expression (< 1%) in hDPSCs (Fig. 2e).

### The expression of BACH1 was changed dynamically during the odontoblastic differentiation of hDPSCs

**Fig. 3:**
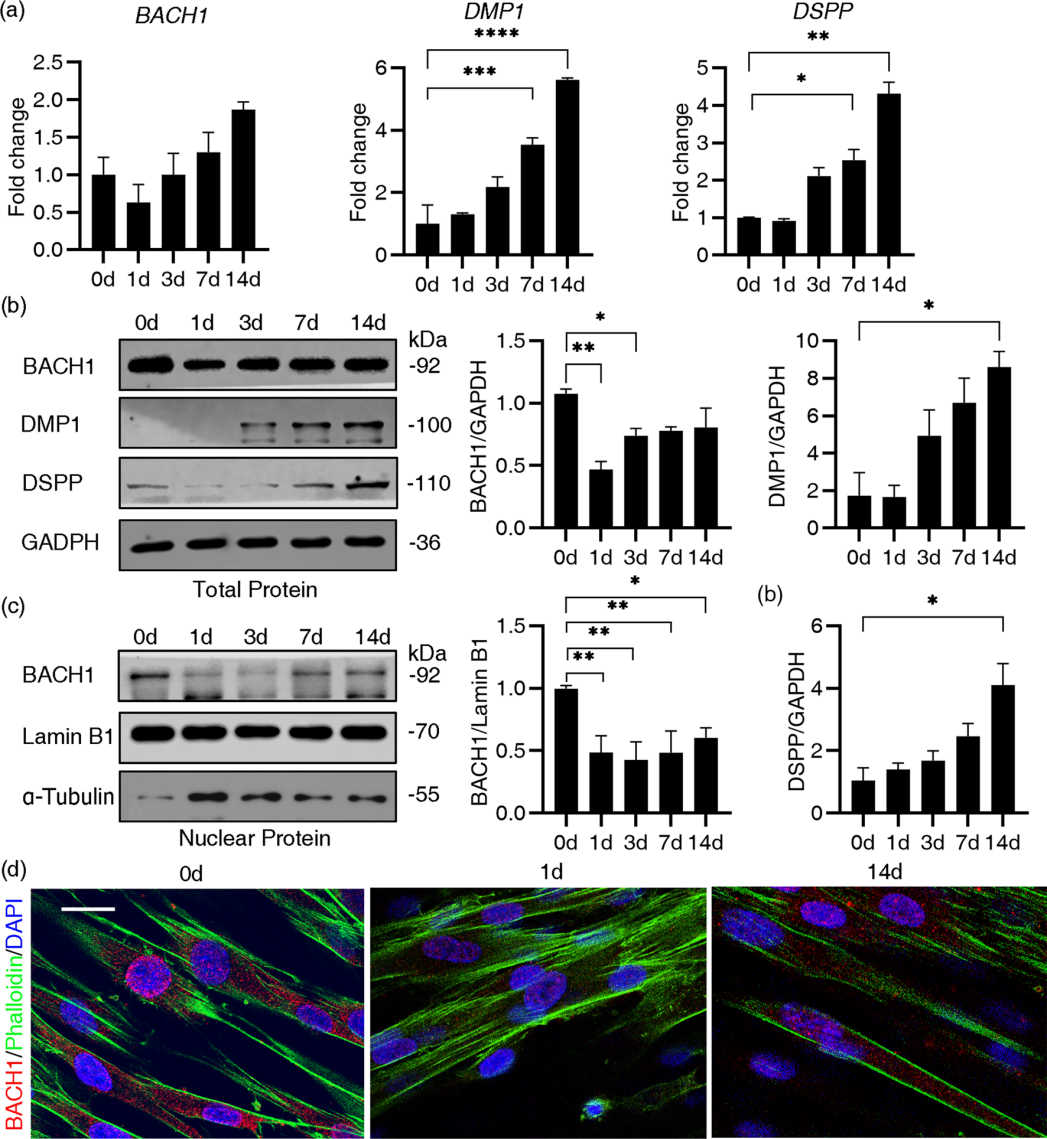
The expression of BACH1 was changed during the odontoblastic differentiation of hDPSCs. **a** Changes in the mRNA level of *BACH1*, *DMP1* and *DSPP* on OM days 0,1,3,7 and 14. *GAPDH* was used as an internal control. **b** Changes in the total protein level of BACH1, DMP1 and DSPP on OM days 0, 1, 3, 7 and 14. Quantification of the relative protein expression via Image Pro Plus software, in which GAPDH was served as the internal control. **c** BACH1 protein expression of nuclear fraction on OM days 0, 1, 3, 7 and 14 had been tested by Western blot analysis. Lamin B1 was used as loading control. **d** Representative immunofluorescent labelling images are displayed. BACH1 (red), DAPI (blue) and Phalloidin (green) were imaged to show the subcellular distribution of BACH1 on OM days 0, 1 and 14. Scale bar = 10 µm. Data were presented as the mean ± standard error of the mean (n = 3). Statistically significant compared to the day 0 group. *P < 0.05, **P < 0.01, ***P < 0.001, ****P < 0.0001. The gels in b, c were cropped, full-length gels are presented in Additional file 1: Original Band of Western blot Analysis Figs. S1 and S2. In Fig. 3b, the bands of BACH1 and GAPDH were from the same gel, while the bands of DSPP and DMP1 were from different gels. In Fig. 3c, the bands of BACH1 and Lamin B1 were from the same gel

We cultured hDPSCs in OM and harvested them at different time points (days 0, 1, 3, 7 and 14). Expression of DMP1 and DSPP (Fig. 3a, b), ALP activity and mineral deposit (Additional file 2: Fig. S1a, b) were tested to confirm the odonto/osteogenic differentiation. The mRNA expression of *BACH1* showed a slight upward trend (Fig. 3a). The protein level of total BACH1 decreased significantly in the first three days and recovered progressively (*P* < 0.05) (Fig. 3b, d). A significant decrease in nuclear BACH1 occurred initially and then gradually increased subsequently, although the protein level on day 14 remained slightly lower than that on day 0 (*P* < 0.05) (Fig. 3c).

### The downregulation of BACH1 impeded the proliferation and induced cell cycle arrest of hDPSCs in vitro

**Fig. 4:**
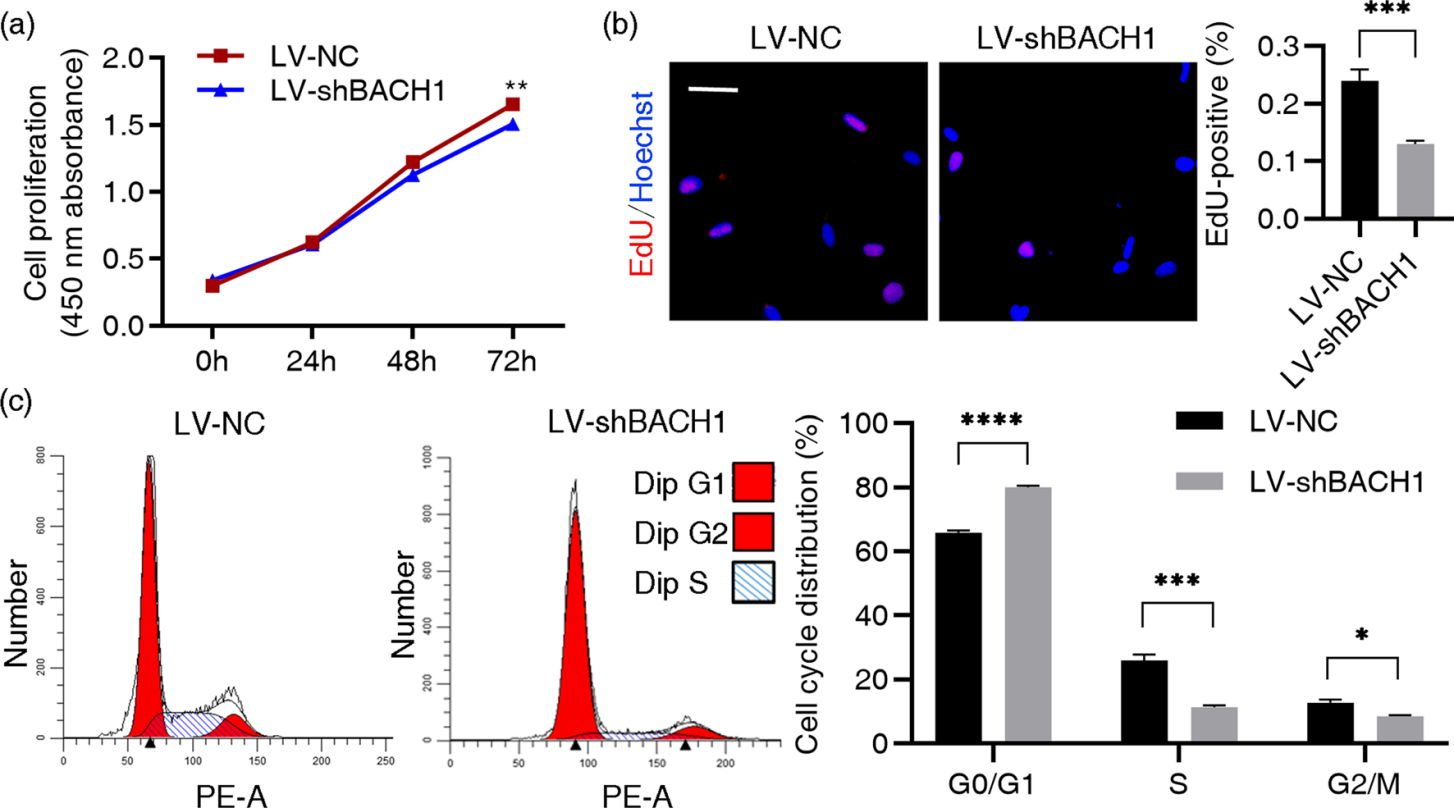
The downregulation of BACH1 impeded the proliferation and induced cell cycle arrest of hDPSCs in vitro. **a** The effect of *BACH1* silencing on the cell proliferation of hDPSCs was determined by CCK-8 assay. *BACH1* silencing group (LV-shBACH1) and control group (LC-NC). **b** EdU assay for cell proliferation. Red, EdU-positive cells; Blue, Hoescht-stained cells. The percentage of EdU-positive cells in the LV-shBACH1 and LC-NC was recorded. Scale bar = 50 µm. **c** Percentage of cell cycle distribution tested by flow cytometry. Statistically significant compared to the LV-NC group. *P < 0.05, **P < 0.01, ***P < 0.001

*BACH1* expression was regulated by using lentiviral vector infection, and the efficiency was tested using qRT-PCR and Western blot analysis (Additional file 2: Fig. S2). The CCK-8 assay revealed that *BACH1*-knockdown significantly impaired the proliferation of hDPSCs (Fig. 4a) while *BACH1-*overexpression increased the proliferation (Additional file 2: Fig. S3a). The EdU assay, one used to identify cells undergoing DNA synthesis, revealed remarkably fewer proliferating (EdU-positive) cells in the LV-shBACH1 group than that of the control group (Fig. 4b). As cells that have DNA replication are in the S phase of the cell cycle, the results of the EdU labelling assay were in agreement with the cell cycle test. The *BACH1*-knockdown group showed a lower proportion of S and G2/M phases and a higher proportion of G0/G1 phases implying cell cycle arrest (Fig. 4c).

### BACH1 affected the odontoblastic differentiation of hDPSCs in vitro

**Fig. 5:**
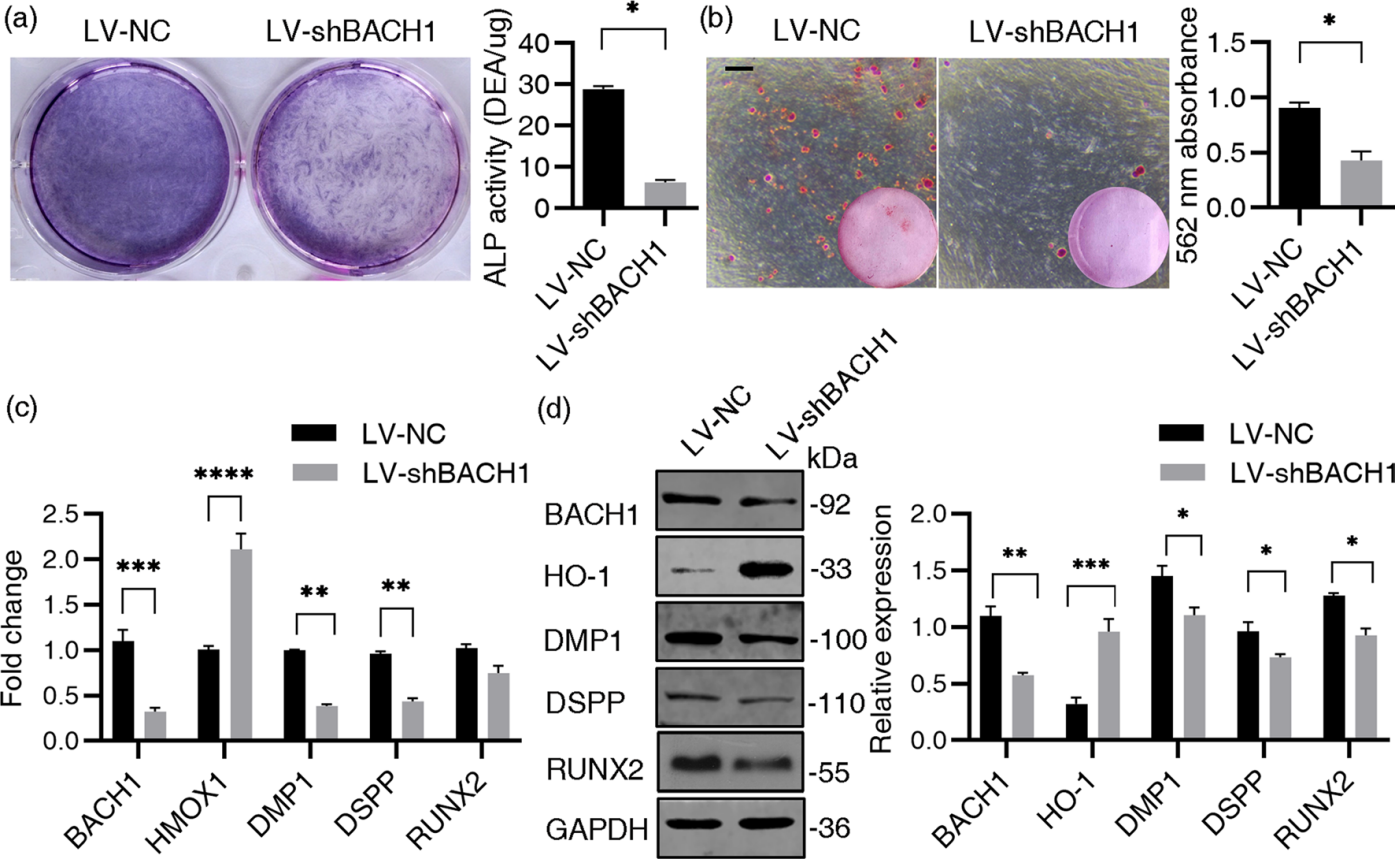
BACH1 affected the odontoblastic differentiation of hDPSCs in vitro. **a** Transduced hDPSCs were cultured with OM for 7 days. ALP activity was evaluated by ALP staining and ALP activity assay in the LV-NC and LV-shBACH1 group. **b** hDPSCs were cultured with OM for 21 days. Calcium nodule deposition was evaluated by ARS staining assay. Scale bar = 200 µm. **c** mRNA level of *BACH1*, *HMOX-1* and odontogenic markers in LV-NC and LV-shBACH1 hDPSCs on OM 14 days. *GAPDH* was used as an internal control. **d** Total protein level of BACH1, HO-1 and odontogenic markers in LV-NC and LV-shBACH1 hDPSCs on OM 14 days. Quantification of the relative protein expression via Image Pro Plus software. GAPDH was used as an internal control. Data were presented as the mean ± standard error of the mean (n = 3). Statistically significant compared to the LV-NC group. *P < 0.05, **P < 0.01, ***P < 0.001, ****P < 0.0001. The gels in d were cropped, full-length gels are presented in Additional file 1 Original Band of Western blot Analysis Fig. S3. The bands of BACH1, DSPP, HO-1 and GAPDH were from the same gel, while the bands of RUNX2 and DMP1 were from different gels

The LV-shBACH1 group displayed impaired ALP staining and decreased ALP activity on day 7 (Fig. 5a), upregulated expression of HO-1, downregulated expression of RUNX2, DMP1 and DSPP on day 14 (Fig. 5c, d) and diminished calcium nodules formation on day 21 (Fig. 5b) compared with the LV-NC group. On the contrary, overexpression of *BACH1* increased ALP activity on day 7 and upregulated the expression level of *DMP1* and *DSPP* on day 14 (Additional file 2: Fig. S3b, c).

### The downregulation of BACH1 impaired the odontoblastic differentiation of hDPSCs was HO-1-independent

**Fig. 6:**
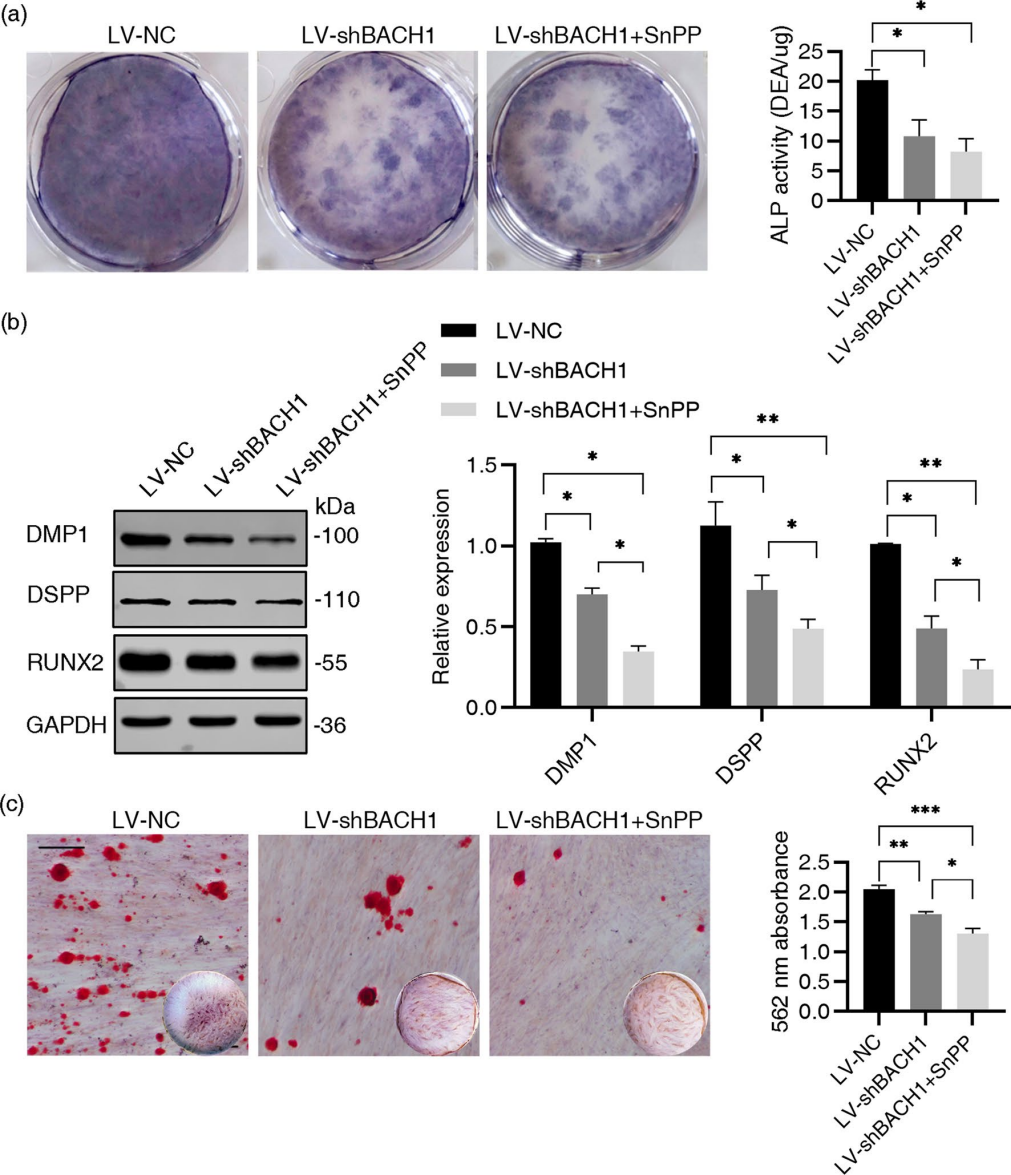
The downregulation of BACH1 impaired the odontoblastic differentiation of hDPSCs was HO-1-independent. **a** Transduced hDPSCs were cultured for 7 days. ALP activity was evaluated by ALP staining and ALP activity assay in the LV-NC, LV-shBACH1 and LV-shBACH1 + SnPP group. **b** Total protein level of odontogenic markers in LV-NC, LV-shBACH1 and LV-shBACH1+ SnPP hDPSCs on 14 days. Quantification of the relative protein expression of odontogenic markers via Image Pro Plus software. GAPDH was used as an internal control. Statistically significant compared to the control group. **c** hDPSCs were cultured for 21 days. Calcium nodule deposition was evaluated by ARS staining assay. Scale bar = 1 mm. SnPP, Tin-protoporphyrin IX. hDPSCS were cultured in OM or OM + SnPP (10 µM). Data were presented as the mean ± standard error of the mean (n = 3). Statistically significant compared to every other group. *P < 0.05, **P < 0.01, ***P < 0.001. The gels in b were cropped, full-length gels are presented in Additional file 1 Original Band of Western blot Analysis Fig. S4. The bands of DSPP, RUNX2 and GAPDH were from the same gel, while the band of DMP1 was from other gel

A significant increase in HO-1 expression was observed in the LV-shBACH1 group compared to the LV-NV group (Fig. 5c, d). To explore whether the effect of BACH1 on the odontoblastic differentiation of hDPSCs is HO-1-dependent, *BACH1*-knockdown hDPSCs were cultured in the OM with Tin-protoporphyrin IX (SnPP, 10 µM), an inhibitor of HO-1. The LV-shBACH1 plus SnPP group displayed impaired ALP staining on day 7, lower protein expression of RUNX2, DMP1 and DSPP on day 14 and less mineral deposit on day 21 compared to the LV-shBACH1 group (Fig. 6). Treatment of SnPP could not restore the decline in differentiation ability but rather exacerbated it. Upregulation of HO-1 partially compensated for the damaged odontoblastic differentiation ability caused by *BACH1*-silence.

## Discussion

A mature tooth is a unique organ composed of non-vascularized hard tissue: enamel, dentin, cementum, and a soft vascularized connective tissue: dental pulp [1]. Induction proliferation, migration and odontoblastic differentiation of stem cells in the human dental pulp is a potential cellular approach for pulp capping or dentin regeneration therapy [8]. hDPSCs have strong self-renewal, proliferation and multiple differentiation abilities [5, 9]. BACH1 is involved in various physiological and pathological processes and participates in the cell differentiation [16, 18, 19, 22]. However, the role of BACH1 in the proliferation and odontoblastic differentiation of hDPSCs has rarely been explored.

BACH1 may be involved in the odontoblastic differentiation of hDPSCs and the homeostasis of the dentin-pulp complex considering its distribution in healthy human pulp tissues and its altered expression patterns during the process of odonto/osteogenic differentiation of hDPSCs. BACH1 is strongly expressed in the odontoblast layer and also in fibroblast-like cells in the cell rich zone of healthy human dental pulp tissues. Odontoblasts are not only involved in the protection of the dental pulp but also in forming mineralized tissue [2]. Additionally, dental pulp fibroblasts exert crucial functions in innate immunity, dental pulp complex regeneration, and tissue homeostasis regulation [8]. During the mineralization process, the protein level of BACH1 undergo a dynamic change, showing a downward and then an upward trend. Treatment with OM decreased of BACH1 at the total protein level but not the mRNA level, suggesting the involvement of post-translational regulation of BACH1. BACH1 was proposed to be regulated by the ubiquitin–proteasome system and can be stabilised by ubiquitin carboxyl-terminal hydrolase 47 and MG132 (Z-Leu-Leu-Leu-al, a proteasome inhibitor) via deubiquitination [23, 24]. The reduction of nuclear BACH1 is mainly attributed to the BACH1 nuclear export which is associated with chromosome region maintenance 1 and phosphorylation of signaling pathways [25–27]. The upregulation of mRNA expression may be the result of a feedback loop of BACH1 to maintain BACH1 equilibrium [26].

To investigate the role of BACH1 in the proliferation and odontoblastic differentiation of hDPSCs, lentiviral vector infection was utilized to remain long-term suppression or overexpression of *BACH1*. The influence of BACH1 on cell proliferation and survival may vary greatly depending on cell type. In primary keratinocytes, *BACH1*-ablation resulted in decreased cell proliferation [28]. However, in human umbilical vein endothelial cells, *BACH1* overexpression inhibited cell proliferation and induced cell-cycle arrest [29]. As for the hDPSCs, *BACH1* down regulation impaired the proliferation ability, possibly due to the involvement of BACH1 in the cell cycle regulation. Knockdown of *BACH1* affected the genes related to cell cycle [15]. Besides, cytosolic BACH1 can interact with hyaluronan-mediated motility receptors to stabilize mitotic spindle orientation and influence mitotic chromosome formation [30].

There are many cytokines engaged in the odontoblastic differentiation of hDPSCs. Alkaline phosphatase, upregulated in the early stages of odontoblast/osteoblast maturation, is an enzyme effective in mineral deposition to enable the formation of hydroxyapatite [31]. RUNX2, involved in the production of bone matrix proteins, is the main molecular regulator responsible for osteogenic differentiation [32]. DMP1 is essential for later dentinogenesis during postnatal development [33]. DSPP is a terminal differentiation marker for the odonto/osteogenic differentiation of hDPSCs [34]. ALP activity on day 7, expression levels of RUNX2, DMP1 and DSPP on day 14, and mineral deposit on day 21 were detected to ensure the effect of induction by OM treatment and measure the degree of odontoblastic differentiation in previous studies [35]. In our study, *BACH1* overexpression elevated the ALP activity and the expression of mineralization markers while *BACH1* knockdown weakened the odontoblastic differentiation of hDPSCs illustrating that BACH1 was required for the odontoblastic differentiation of hDPSCs in vitro. It has been reported that downregulation of BACH1 protected murine osteoblast-like cell line MC3T3-E1 against hydrogen peroxide (H_2_O_2_)-induced oxidative damage and preserved osteoblast differentiation in hyperoxic condition [36]. The opposite results may due to the different induction conditions.

As the decline of nuclear BACH1 could relieve its inhibition effect on *HMOX1* gene promoter [37], *BACH1*-knockdown induced higher expression of HO-1 during the mineralization of hDPSCs and odontoblasts express BACH1 in cytoplasm at high levels along with high expression of HO-1. Whether the cytoplasmic retention of BACH1 only means the regeneration and regulation of BACH1 or it has other effects need to be further studied. The role of HO-1 in odonto/osteogenic differentiation is quite controversial. Lin et al. reported that upregulation of HO-1 inhibited the differentiation and maturation of osteoblasts, which was reversed by treatment of an HO-1 inhibitor [38]. However, administration of an HO-1 inhibitor further diminished the odontoblastic differentiation ability of *BACH1*-knockdown hDPSCs. Our findings were consistent with previous discovery that HO-1 expressed in the odontoblast layer highly and affected odontoblastic differentiation positively [39, 40]. In other words, BACH1-dificiency impaired the odontoblastic differentiation of hDPSCs in an HO-1-independent manner. Knocking down *BACH1* in human cells can influence several HO-1-independent signaling pathways, including phosphoinositide 3-kinase/protein kinase B signaling pathway [41], mitogen-activated protein kinase signaling pathway [41, 42], and the Wnt/β-catenin signaling pathway [22], all of which are associated with odonto/osteogenic differentiation of hDPSCs [43, 44]. Previous studies exploring the targets of BACH1 give us the inspiration for the next step to explore the mechanism and might contribute to our result that silencing of BACH1 breaks up the odontoblastic differentiation of hDPSCs. Future research will need to clarify how BACH1 functions in the network that controls hDPSCs differentiation.

## Conclusions

The current study provided evidence that downregulation of BACH1 inhibited the proliferation and impeded odontoblastic differentiation of hDPSCs whereas upregulation of BACH1 could increase cell proliferation, ALP activity and the expression of mineralization markers. These findings support that BACH1 might positively affect the ability of hDPSCs to promote regenerative or reparative processes.

## Supplementary Information

**Additional file 1:** Original band of western blot analysis.

**Additional file 2:** Supplementary materials.

## Data Availability

All data generated or analyzed during this study are included in this published article and its Additional files 1 and 2.

## Abbreviations

α-MEM: Alpha minimum essential medium
ALP: Alkaline phosphatase
ARS: Alizarin red S
BACH1: BTB and CNC homology 1
CCK-8: Cell counting kit-8
DAPI: 4,6-Diamidino-2-phenylindole
DMP1: Dentin matrix protein 1
DSPP: Dentin sialophosphoprotein
EdU: 5-Ethynyl-2'-deoxyuridine
FBS: Foetal bovine serum
GAPDH: Glyceraldehyde 3-phosphate dehydrogenase
H&E: Hematoxylin–eosin
hDPSCs: Human dental pulp stem cells
HO-1: Heme oxygenase-1
OM: Odonto/osteogenic induction medium
RUNX2: Runt-related transcription factor 2
SnPP: Tin-protoporphyrin IX
TBST: Tris-buffered saline with 0.05% Tween-20
