# Supplementary material for: BACH1 regulates the proliferation and odontoblastic differentiation of human dental pulp stem cells
Source: BMC Oral Health. 2022 Nov 24;22:536. doi: 10.1186/s12903-022-02588-2 (PMC9694919; doi:10.1186/s12903-022-02588-2)
Supplement: Supplementary file 1 — Additional file 1: Original band of western blot analysis. [file 12903_2022_2588_MOESM1_ESM.pdf]

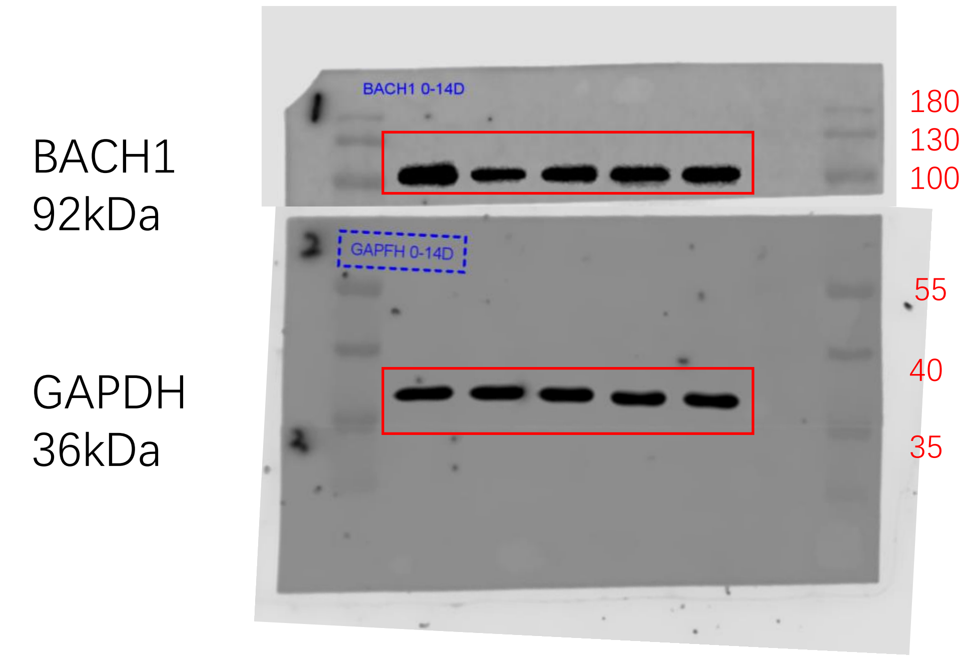

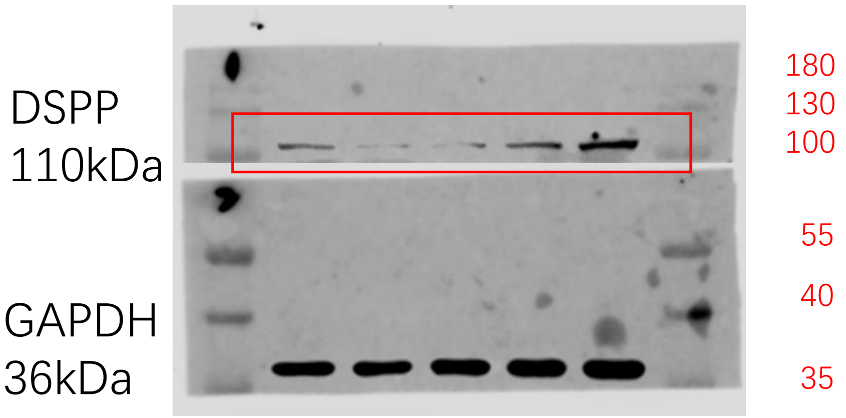

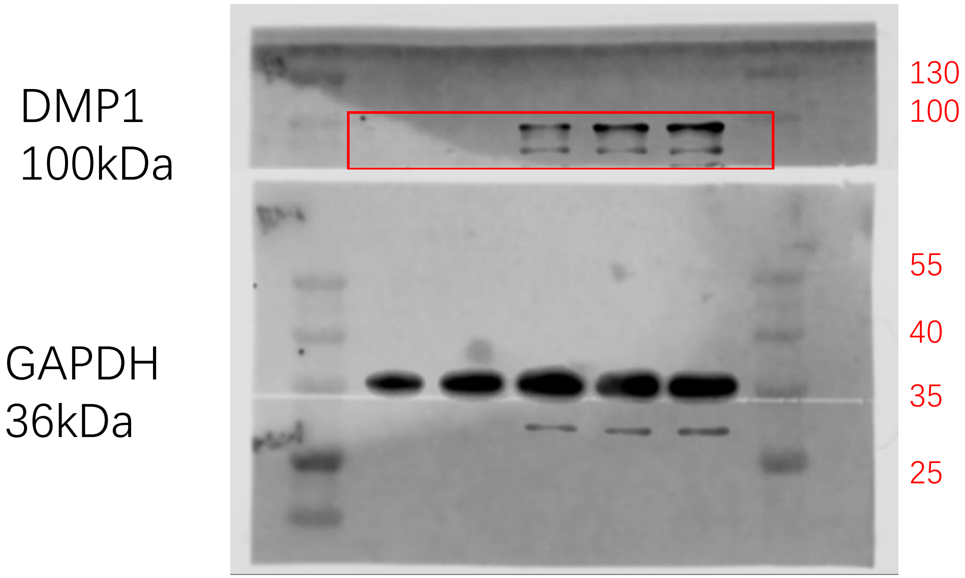

Supplementary Figure 1: Manuscript related files Fig 2b. The BACH1, GAPDH protein bands are corresponding the original band of Fig. 2b in the manuscript. The red box shows the part of the gels used in the manuscript. The target protein bands we selected should be along with the internal control.

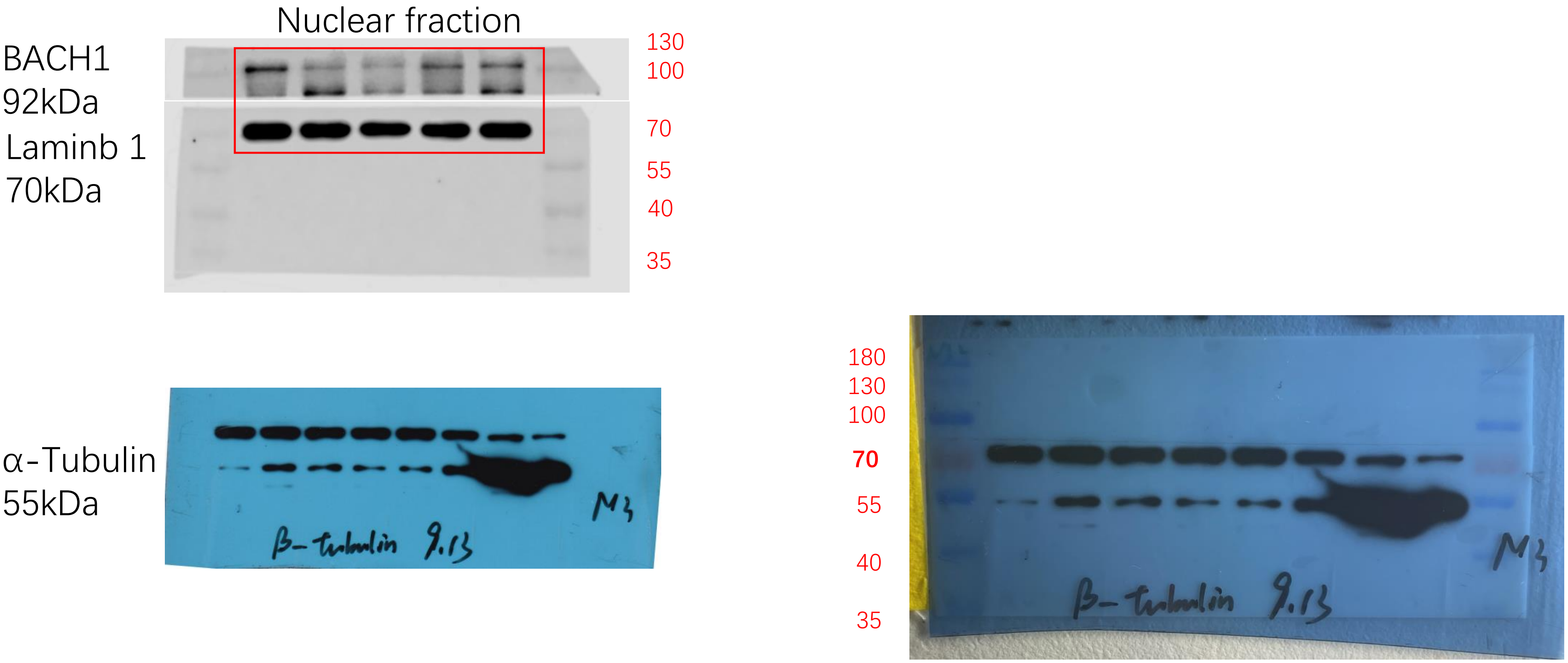

Supplementary Figure 2: Manuscript related files Fig 2c. The BACH1,Lamin B1, α-Tubulin protein bands are corresponding the original band of Fig. 2c in the manuscript. The red box shows the part of the gels used in the manuscript. The target protein bands we selected should be along with the internal control.

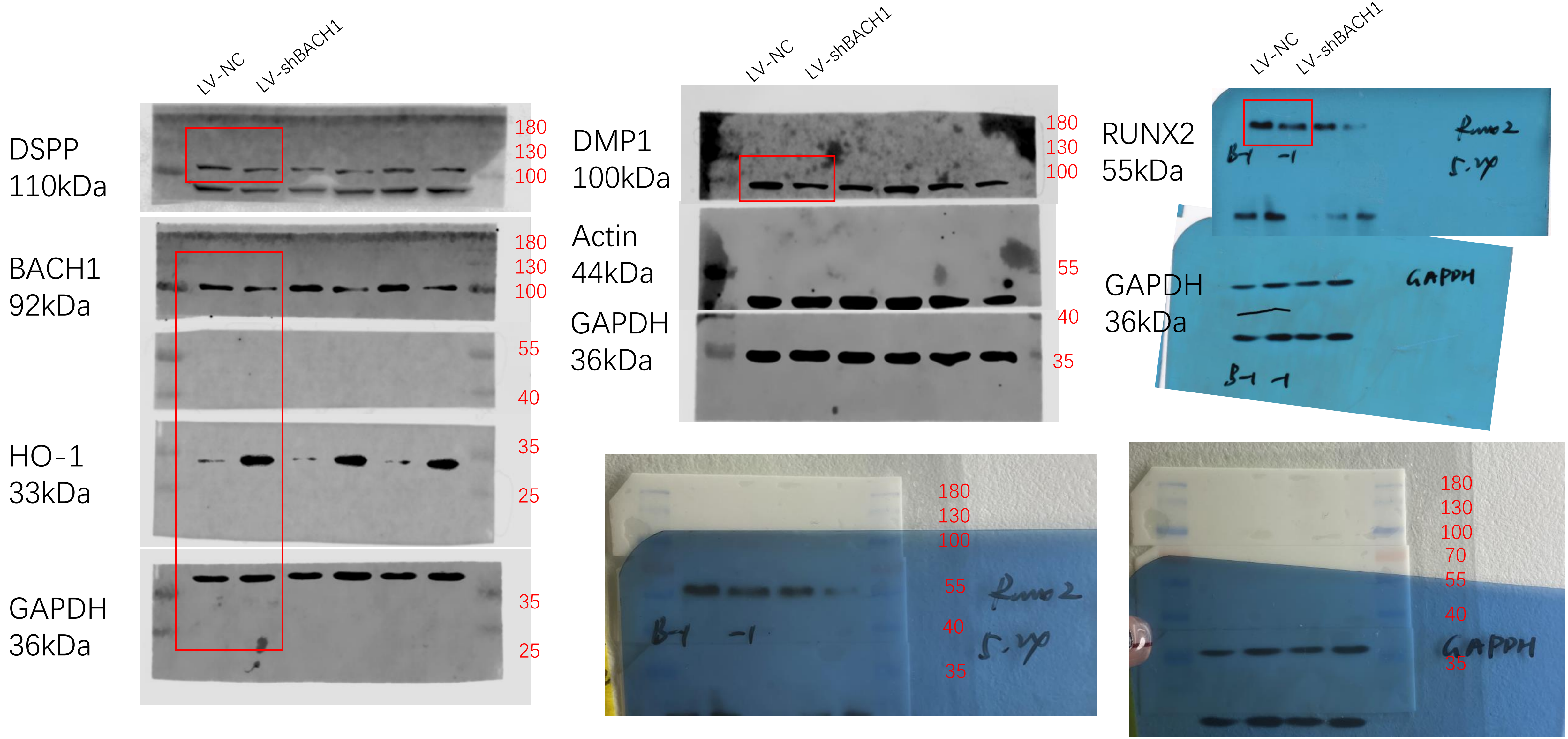

Supplementary Figure 3: Manuscript related files Fig 4d. The BACH1, DMP1, DSPP, RUNX2, GAPDH protein bands are corresponding the original band of Fig. 4d in the manuscript. The red box shows the part of the gels used in the manuscript. The target protein bands we selected should be along with the internal control.

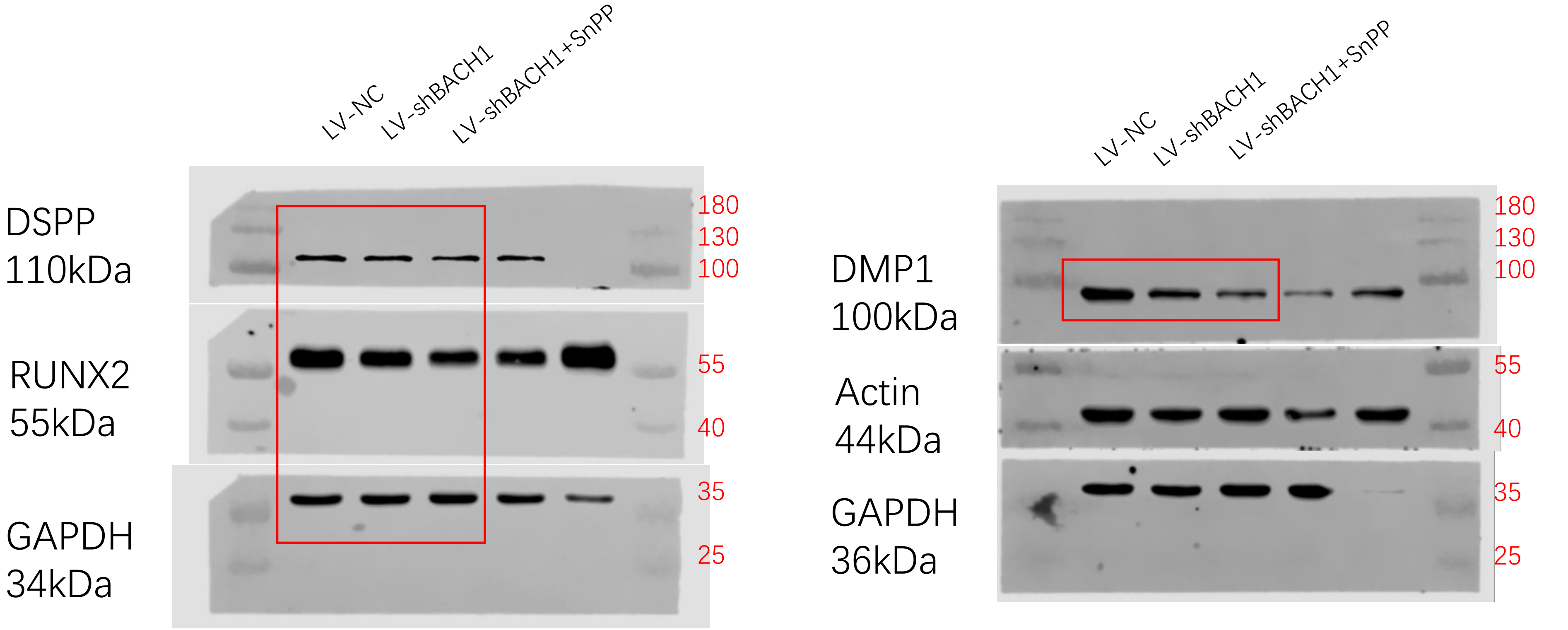

Supplementary Figure 4: Manuscript related files Fig 5b. The DMP1, DSPP, RUNX2, GAPDH protein bands are corresponding the original band of Fig. 5b in the manuscript. The red box shows the part of the gels used in the manuscript. The target protein bands we selected should be along with the internal control.

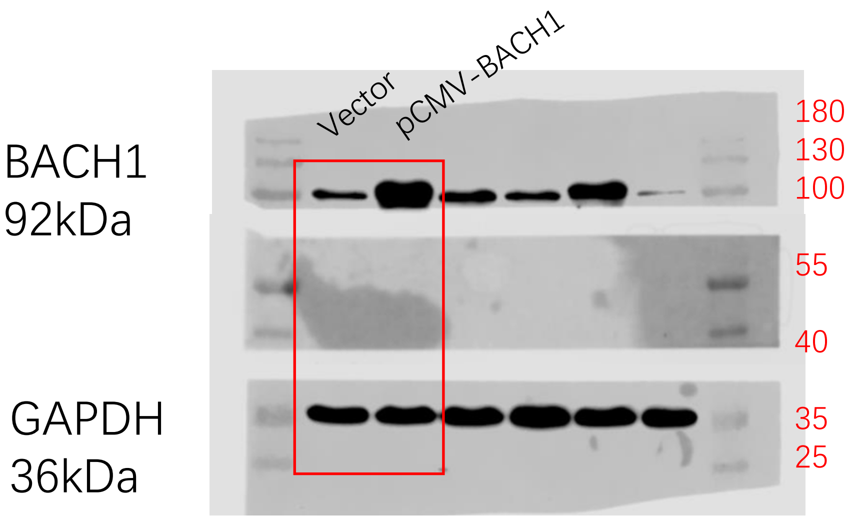

BACH1 overexpression efficiency

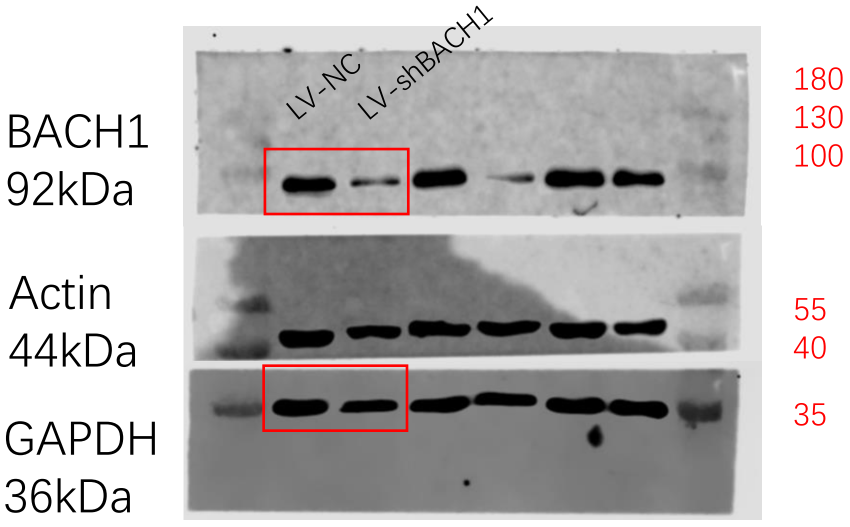

BACH1 Knock down effeciency

Supplementary Figure 5: Supplementary related files Fig 2c. The BACH1, GAPDH protein bands are corresponding the original band in Supplementary material Fig 2c. The red box shows the part of the gels used in the manuscript. The target protein bands we selected should be along with the internal control.
