# Supplementary material for: BACH1 regulates the proliferation and odontoblastic differentiation of human dental pulp stem cells
Source: BMC Oral Health. 2022 Nov 24;22:536. doi: 10.1186/s12903-022-02588-2 (PMC9694919; doi:10.1186/s12903-022-02588-2)
Supplement: Supplementary file 2 — Additional file 2: Supplementary materials. [file 12903_2022_2588_MOESM2_ESM.docx]

**BACH1 regulates the proliferation and odontoblastic differentiation of human dental pulp stem cells**

**Supplementary Table 1** Components of adipogenic differentiation maintenance medium

| Kit components | 200 mL/kit |
| --- | --- |
| Adipogenic Differentiation Induction Basal Medium | 175 mL |
| Adipogenic Differentiation FBS | 20 mL |
| P/S Solution | 2 mL |
| Glutamine | 2 mL |
| Insulin | 400 μL |

| **Supplementary Table 2** Components of adipogenic differentiation induction mediumKit components | 200 mL/kit |
| --- | --- |
| Adipogenic Differentiation Induction Basal Medium | 175 mL |
| Adipogenic Differentiation FBS | 20 mL |
| P/S Solution | 2 mL |
| Glutamine | 2 mL |
| Insulin | 400 μL |
| IBMX | 200 μL |
| Rosiglitazone | 200 μL |
| Dexamethasone | 200 μL |

**Supplementary Table 3** Components of chondrogenic differentiation medium

| Kit components | 100 mL/kit |
| --- | --- |
| Chondrogenic differentiation basal medium | 97 mL |
| Ascorbate Acid | 1 mL |
| Sodium Pyruvate | 300 μL |
| Proline | 100 μL |
| Dexamethasone | 100 μL |
| TGF-β3 | 1 mL |

**Supplementary Table 4** Primer sequences for qRT-PCR

| Gene | | Primer sequence（5’-3’） |
| --- | --- | --- |
| *BTB domain and CNC homolog 1* (*BACH1*)  *Heme oxygenase-1*  (*HMOX1*)  *Dentin sialophosphoprotein* (*DSPP*)  *Dentin-matrix protein 1*  (*DMP1*)  *Runt-related transcription factor* 2 (*RUNX2*)  *Glyceraldehyde 3-phosphate*  *dehydrogenase* (*GAPDH*) | Forward  Reverse  Forward  Reverse  Forward  Reverse  Forward  Reverse  Forward  Reverse  Forward  Reverse | TCTGAGTGAGAACTCGGTTTTTG  CGCTGGTCATTAAGGCTGAGTAA  GTCAGGCAGAGGGTGATAGAAG  GTGTAAGGACCCATCGGAGAAG  ATATTGAGGGCTGGAATGGGGA  TTTGTGGCTCCAGCATTGTCA  AGACAGTGCCCAAGATACCACC  ATTCCCTCATCGTCCAACTCG  TCCACACCATTAGGGACCATC  TGCTAATGCTTCGTGTTTCCA  TTCTTTTGCGTCGCCAGCCGA  GTGACCAGGCGCCCAATACGA |


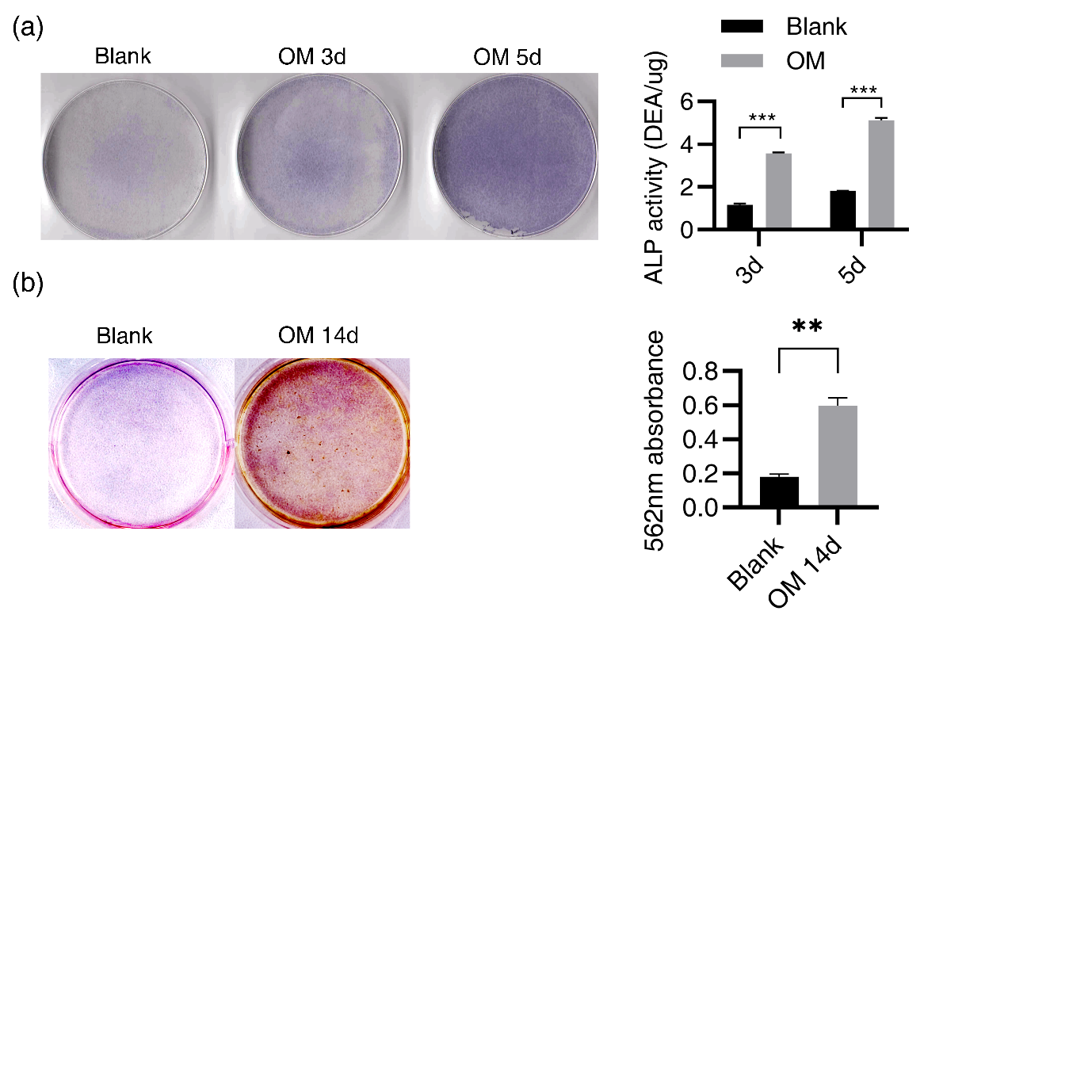


**Supplementary Figure 1.** (a) hDPSCs were cultured in OM for 3 or 5 days. ALP activity was evaluated by ALP staining and ALP activity assay. (b) hDPSCs were cultured in OM for 14 days. Calcium nodule deposition was evaluated by Alizarin red S staining assay. Statistically significant compared with the control group. ***P* < 0.01, ****P* < 0.001.


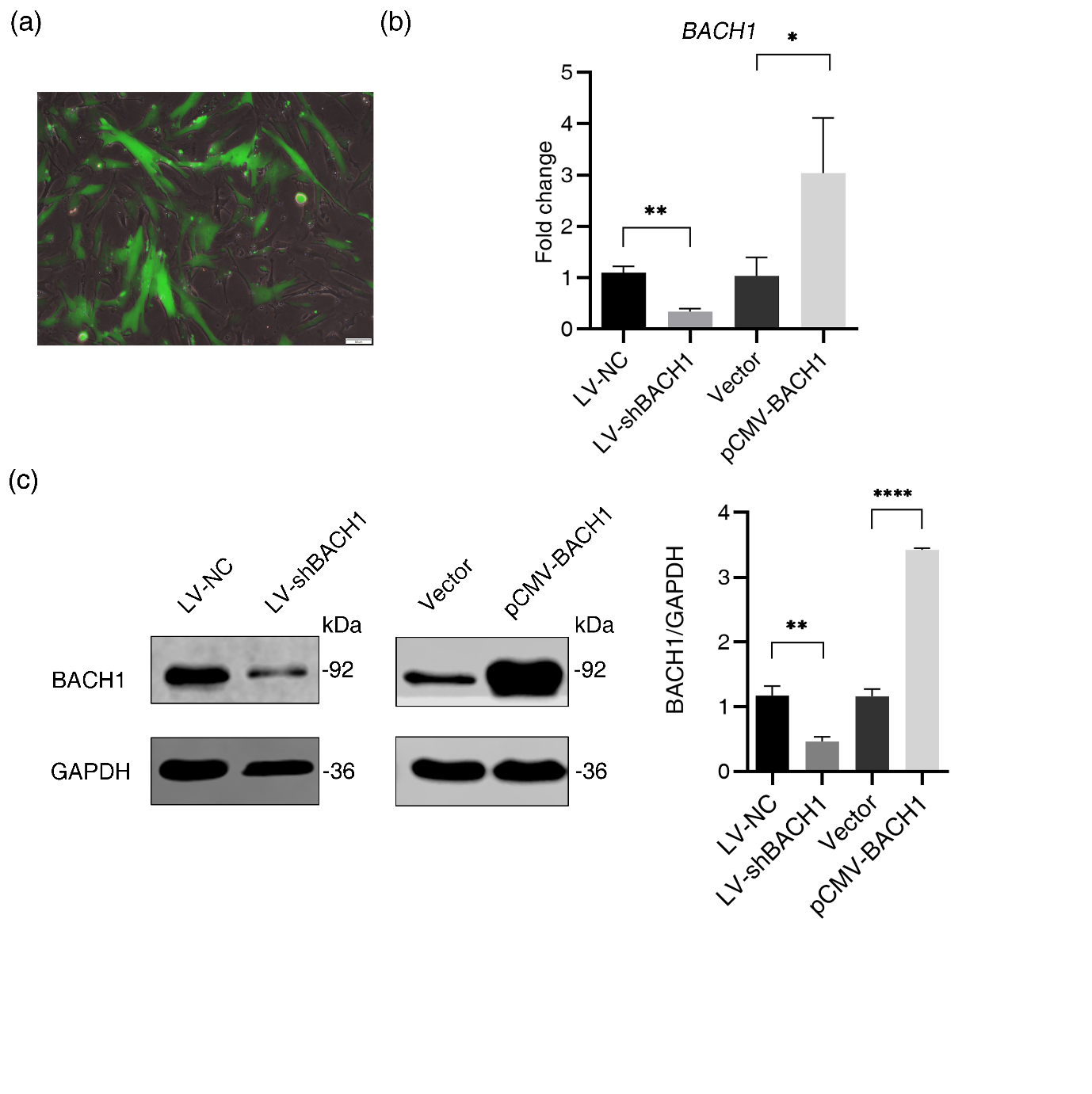


**Supplementary Figure 2.** (a) Transfected hDPSCs grew well at 72h after gene transduction, and green fluorescent protein (GFP) fluorescence in hDPSC confirmed transfection efficiency. (b) The mRNA expression level of *BACH1.* *GAPDH* was used as an internal control. (c) The protein expression level of BACH1. GAPDH was used as an internal control. Scale bar = 50 μm. Statistically significant compared with the control group. **P* < 0.05, ***P* < 0.01.


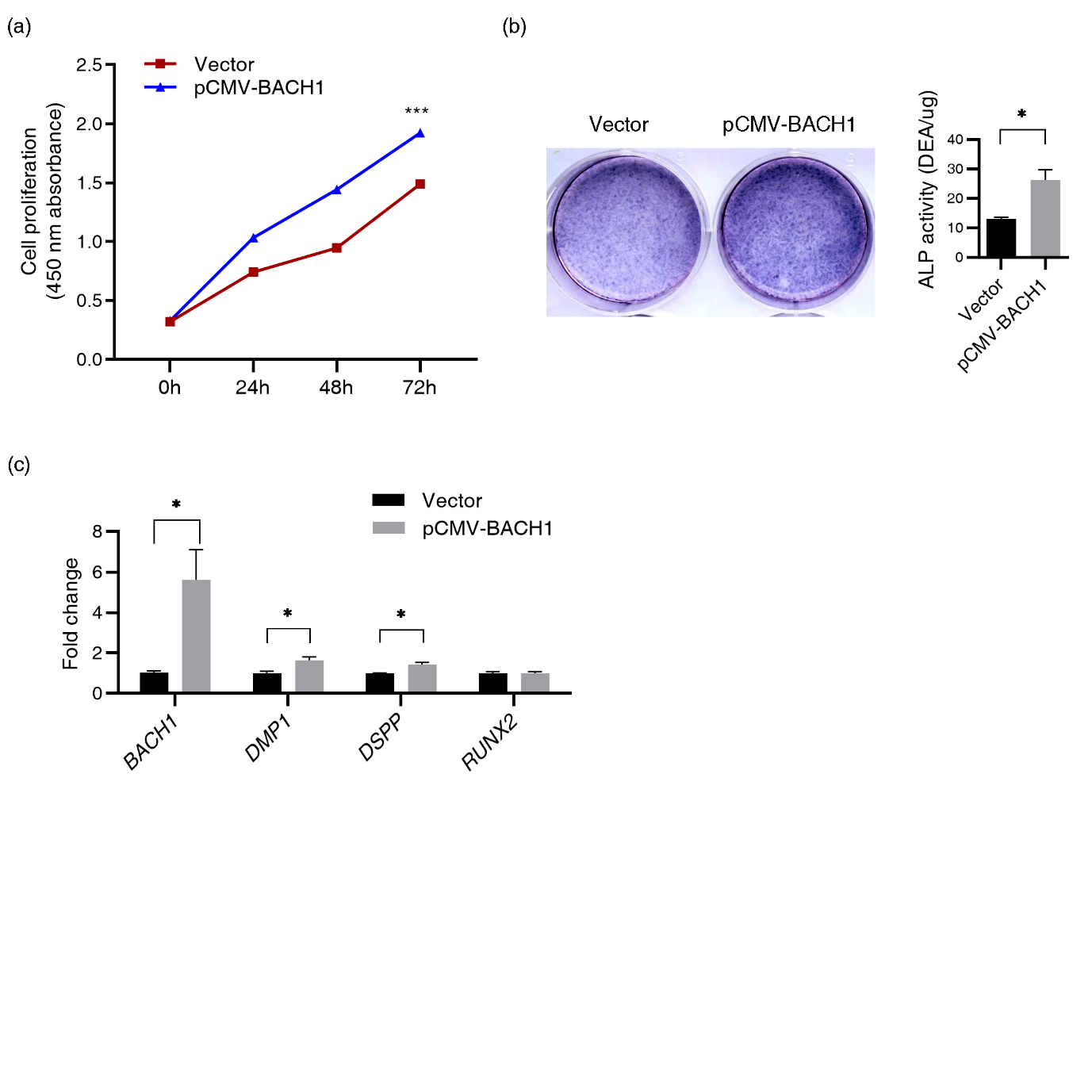


**Supplementary Figure 3.** (a) The effect of BACH1 upregulating on the cell proliferation of hDPSCs was determined by CCK-8 assay. BACH1 overexpression group (pCMV-BACH1) and control group (Vector). (b) Transduced hDPSCs were cultured with OM for 7 days. ALP activity was evaluated by ALP staining and ALP activity assay in the Vector and pCMV-BACH1 group. (c) mRNA level of *BACH1* and odontogenic markers in in the Vector and pCMV-BACH1 hDPSCs on OM 14 days. *GAPDH* was used as an internal control. **P* <0.05, ***P* < 0.01, ****P* < 0.001.
